# A trial of a job-specific workers' health surveillance program for construction workers: study protocol

**DOI:** 10.1186/1471-2458-11-743

**Published:** 2011-09-29

**Authors:** Julitta S Boschman, Henk F van der Molen, Cor van Duivenbooden, Judith K Sluiter, Monique HW Frings-Dresen

**Affiliations:** 1 Academic Medical Center, University of Amsterdam, Department: Coronel Institute of Occupational Health, Amsterdam, the Netherlands; 2 Arbouw, Dutch Health & Safety Institute in the Construction Industry, Harderwijk, The Netherlands

## Abstract

**Background:**

Dutch construction workers are offered periodic health examinations. This care can be improved by tailoring this workers health surveillance (WHS) to the demands of the job and adjust the preventive actions to the specific health risks of a worker in a particular job. To improve the quality of the WHS for construction workers and stimulate relevant job-specific preventive actions by the occupational physician, we have developed a job-specific WHS. The job-specific WHS consists of modules assessing both physical and psychological requirements. The selected measurement instruments chosen, are based on their appropriateness to measure the workers' capacity and health requirements. They include a questionnaire and biometrical tests, and physical performance tests that measure physical functional capabilities. Furthermore, our job-specific WHS provides occupational physicians with a protocol to increase the worker-behavioural effectiveness of their counselling and to stimulate job-specific preventive actions. The objective of this paper is to describe and clarify our study to evaluate the behavioural effects of this job-specific WHS on workers and occupational physicians.

**Methods/Design:**

The ongoing study of bricklayers and supervisors is a nonrandomised trial to compare the outcome of an intervention (job-specific WHS) group (n = 206) with that of a control (WHS) group (n = 206). The study includes a three-month follow-up. The primary outcome measure is the proportion of participants who have undertaken one or more of the preventive actions advised by their occupational physician in the three months after attending the WHS. A process evaluation will be carried out to determine context, reach, dose delivered, dose received, fidelity, and satisfaction. The present study is in accordance with the TREND Statement.

**Discussion:**

This study will allow an evaluation of the behaviour of both the workers and occupational physician regarding the preventive actions undertaken by them within the scope of a job-specific WHS.

**Trial registration:**

NTR3012

## Background

Dutch construction workers are offered periodic health examinations in order to detect risk factors for work-related diseases and changes in health. If necessary, preventive actions can be taken to prevent deterioration of their health status and to improve work functioning. This care can be improved by tailoring workers' health surveillance (WHS) to the demands of the job and adjusting the intervention measures to the specific needs associated with particular jobs. To improve the quality of the WHS for construction workers and to increase recommendations of job-specific intervention measures by occupational physicians (OPs), we developed a job-specific WHS. The rationale for developing job-specific WHS for construction workers is based on the consideration that the aims and content of the WHS and proposed interventions should be tailored to the occupation of interest.

The goals of WHS are as follows: (i) to prevent the onset, recurrence and/or worsening of work-related diseases; (ii) to monitor and promote individuals' health in relation to work; and (iii) to monitor and promote work functioning and deployment [1]. Accordingly, each WHS is designed in such a way that it is relevant to the nature of the demands and health effects of the occupation of interest [2]. Evidence from the literature supports the perception that a bricklayer has a physically demanding occupation whereas the construction supervisor has a mentally demanding occupation. A systematic review of the literature revealed details on the work demands and health effects of both occupations [3]. These details suggest that a fruitful approach would be to develop and implement a job-specific WHS for construction occupations aimed at the demands and health effects associated with the work.

The screening instruments selected for the WHS should be attuned to the physical and psychological requirements of the job. Several authors have argued the relevance of job-specific measurement instruments. For example, Tufts et al. [4] explicate the need for tests measuring auditory fitness for duty. Other examples are the trucker strain monitor for measuring psychological job strain in truck drivers [5,6], a functional capacity evaluation for hospital nurses [7] and the fire fighting simulation test for fire fighters [8]. The current instruments used in the Dutch WHS for construction workers are lacking such an approach.

A job-specific WHS should incorporate job-specific (and preferably evidence-based) intervention measures. A job-specific occupational health education program for preventing work-related musculoskeletal back injuries in construction labourers, for example, has proven to be effective [9]. Other examples of recommendable job-specific interventions for bricklayers are the use of a scaffolding console to reduce the frequency and duration of trunk flexion during bricklaying [10,11] and the use of mechanised transport to reduce the frequency of handling objects [10]. A structured protocol for advising and counselling OPs on job-specific intervention measures does not exist in the Netherlands. Such a protocol could facilitate the recommendation of preventive actions by OPs.

In the present paper, we refer to the bricklayers and supervisors participating in the study as 'workers', in order to distinguish them from the other participants in the trial: OPs, ergonomists and physician's assistants.

### Objectives

We hypothesise that the job-specific WHS will influence workers who attend it to undertake preventive actions. Moreover, we hypothesise that based on the job-specific WHS, OPs will advise more preventive actions in total and more job-specific (compared with general health) preventive actions. The objective of this paper is to describe the study protocol used to evaluate the job-specific WHS and to test these hypotheses.

## Methods/design

### Dutch context

In the Netherlands, employers are obliged to offer their employees a medical examination, as stated in Article 18 of the Dutch Working Circumstances and Occupational Health and Safety Act [12]. Employees in the construction industry are invited for such a medical examination once in every four years (when they are under the age of 40) or every two years (when they are over 40 years old). This examination is part of their collective labour agreement. Arbouw (the Health and Safety Institute for the Dutch construction industry) provides this service for all employees in the construction industry. Employees are invited to an Occupational Health Service (OHS) near their place of residence. Attending the periodic medical examination is voluntarily. At the time this study was prepared, the attendance rate was 47% [13]. In the collective labour agreement of the workers is recorded that 1) research is performed at the OHS under the authority of Arbouw and 2) the information collected during the medical examinations can be used for research purposes. When attending their medical examination the workers are reminded in writing of this agreement and they can choose whether or not to participate voluntarily. The workers are aware of the fact that by participating they give the researchers written consent to use their information anonymously for research purposes.

### Study design

In the following description of the design of this study we follow the TREND Statement [14]. The primary objective of the study is to compare how attending a job-specific WHS versus the current WHS (care as usual, CAU) influences the preventive actions undertaken by bricklayers and supervisors (hereafter referred to as 'workers'). Our hypothesis is that the job-specific WHS helps bricklayers and supervisors undertake preventive actions.

Offices of one OHS participate in the study. At the offices of the OHS in the control group, the workers attend CAU. The OPs in the control group counsel their workers according to CAU. Workers in the intervention group attend the job-specific WHS, and the OPs in the intervention group counsel their workers according to the job-specific protocol. After inclusion and baseline measurements, measurements take place at two and three months after baseline. The medical ethics committee of the institution, the Academic Medical Center, approves the present study, and the board of the participating OHS advises positively about the local feasibility of the study.

Blinding of the OPs, physician's assistants, ergonomists or workers is not feasible for several reasons. The workers are familiar with CAU as they are invited regularly (every four or two years) for their WHS. Any modification to the standard procedure would be noticed. Moreover, every OP is trained to apply either the experimental intervention (job-specific WHS) or CAU. Therefore, all the OPs know which type of intervention they perform. To prevent contamination between the two groups, the OPs in the control group will neither be informed about nor receive training in the job-specific WHS. The OPs in the intervention group are requested not to disclose the new intervention during the length of the study to the OPs in the control group. Furthermore, the chance of contamination is small as the OPs in the intervention group work at different offices than the control group. The physician's assistants will be informed about the procedural changes in the job-specific WHS compared to CAU. The ergonomists will be trained to apply the experimental intervention only, as they do not contribute to the CAU.

### Study population and recruitment

The research population includes all workers with the occupation bricklayer or supervisor who are invited by the OHS. Workers are eligible to participate when they are i) primarily working as a bricklayer or construction supervisor; ii) male; iii) able to read, speak and write Dutch sufficiently well and iv) not planning to leave their occupation in the three months after inclusion due to resignation or (early) retirement. Because both occupations consist primarily of men, we include only men.

The study population is recruited through the participating OHS. The workers, who are invited at the offices where the job-specific WHS is implemented, are informed by a letter about the innovation at their OHS. Then, concurrent procedures are performed for both the control and intervention groups: all workers are asked to confirm their attendance, and if necessary, another date or time is arranged. When a worker does not show up without cancelling, the OHS will try to follow-up by phone to make a new appointment if desired by the worker.

### Recruitment of occupational physicians

Participating OPs are recruited between October and December 2011 from offices of the same OHS. These offices are geographically widespread over the Netherlands and were selected on the basis of i) the number of bricklayers and supervisors expected to be invited for a WHS in 2012 and, for the intervention group, ii) the possibility of setting up a job-specific WHS. To provide a safe testing situation, the office must have a covered accommodation of approximately 15 × 15 m and the ability to set a 7 m ladder up against the building.

### Intervention

The Dutch Guideline for Workers' Health Surveillance for the Occupational Physician [1] was followed in the process of developing the job-specific workers' health surveillance (WHS) program. The content of the job-specific WHS was determined in a step-by-step procedure partially based on Bos et al. [15]. The job-specific WHS aims at detecting signals of work-related health problems, reduced work capacity and/or reduced work functioning. The worker fills in a job-specific questionnaire consisting of validated and reliable screening instruments, then biometry measurements are carried out by a physician's assistant, and subsequently the worker performs physical performance tests under the guidance of an ergonomist. The OP uses a structured protocol to assess the results and to prepare the consultation with the worker. Thereafter, the OP discusses the results of the job-specific WHS and recommends desirable preventive actions to the worker in a 20-minute consultation. Together with the worker, a course of action is drawn up and a follow-up appointment is arranged within eight weeks after the job-specific WHS, if necessary. A structured intervention protocol for the OP facilitates job-specific intervention measures and an evaluation of the actions undertaken by the worker. The worker receives a report with the advises of the OP. In the job-specific WHS the following domains are represented: musculoskeletal system, safety (vision, perception of sound, psychological vigilance and working at heights), hazardous substances (skin, lungs), health in relation to work (cardiometabolic health) and work ability.

In Table 1, an overview of the domains and content of the job-specific WHS for both occupations is given. The development and the exact content of the job-specific WHS is described in Additional file 1.

**Table 1:** Overview of the requirements and content of the job-specific workers' health surveillance programs for the two occupations.

Requirements	Instruments
**Physical requirements**	Signalling questions and physical performance tests that -- when possible -- measure combined physical requirements:

*both occupations:*	*both occupations:*
Climbing stairs	Climbing stairs (2 × 6 times)
Climbing a ladder/scaffold	Climbing a ladder (7 m)

*bricklayers only:*	*bricklayers only:*
Standing	Signalling questions hand-arm vibration syndrome [19]
Repetitive movements	Bricklaying test (mixing a mortar-like substance, repetitive hand-arm movements with a trowel, picking up and laying bricks at several heights, lifting and carrying 25-kg sacks)
Working above shoulders	
Working with a bent back	
Squatting and kneeling	
Lifting and carrying (25 kg)	

*supervisors only:*	*supervisors only:*
Walking	Walking 10 min

**Safety requirements**	

Hearing	*both occupations:*
	Audiogram [20]

Eyesight	Signalling questions and eye test at 5 m [21]

Working at heights	Signalling questions (weariness, alertness)

Psychological vigilance	*both occupations:*
	Screening questionnaires:
	Need for recovery after work [22,23]
	Distress [24]
	Depression [25,26]
	Anxiety [25,26]
	Post-traumatic stress disorder [27]
	Risky alcohol consumption [28]
	*supervisors only:*
	Screening questionnaire burn-out [29]

**Health requirements**	

**Working with hazardous substances**	

Skin	*both occupations:*
	Signalling questions skin
	*bricklayers only:*
	Screening questionnaire hand dermatitis [30]

Lungs and airways	*both occupations:*
	Signalling questions skin, lungs and airways [31]
	Spirometry [31]
**Cardiometabolic health**	*both occupations:*
	Risk score cardiometabolic diseases [32]
	Cardiovascular risk profile [33]

**Work functioning**	*both occupations:*
	First 5 questions of the work ability index [34-36]
	*supervisors only:*
	Job-specific addition with mental requirements

### Care as usual

CAU aims at detecting signals of work-related health problems and risk factors. The worker fills in a questionnaire and a physician's assistant carries out biometry measurements. The biometry measurements of both the job-specific WHS and CAU are identical. The questionnaire covers several topics including health, perceptions of work and risk factors at work. Neither scale scores nor cut-off points are calculated, except for work ability. The questionnaire used in CAU is identical for all occupations, and no protocol exists for analysing the results. The OP discusses the results of both the biometry and the questionnaire with the worker in a 20-minute consultation. No structured protocol exists to facilitate the OP in their counselling. A report of the results is sent to the home address of the worker afterwards.

### Training OPs and ergonomists

The OPs and ergonomists who carry out the job-specific WHS participate in a half-day training course. This training course consists of an explanation of the background and development of the job-specific WHS. OPs and ergonomists will be instructed on their protocol to be followed in the WHS and the role of the ergonomist in the WHS.

We consider the procedures and guidelines imposed by Arbouw for carrying out the WHS as care as usual. However, from the pilot study, it became clear that several barriers and ignorance exist regarding these procedures. Therefore, the OPs in the control group receive a two-hour class in order to train them to carry out the WHS according to the guidelines and procedures required by Arbouw.

#### Sample size and assignment

The sample size is based on detecting a difference in activities or interventions undertaken by the worker in the six months after they have attended their WHS. No information on this is available from literature. From the registered activities and interventions in the Arbouw-database, we expect 10% of the workers in the control group to undertake an activity registered by the OP. We have decided that an absolute increase of 20% in interventions undertaken by the workers is a relevant difference. For an increase of 20% (from 10 to 30%) with alpha 0.05 (two-tailed) and a power of (1-beta) = 0.80, power calculation using the Nquery Advisor software results in 75 workers per group. However, we expect a small effect at the level of the OP (ICC = 0.01). Every OP will treat ~15 workers. This results in a design effect of 1.14 ((1+(N-1)*ICC) and an adjusted sample size of 86 per group, divided over at least 6 OPs [16]. With an expected loss to follow-up of 20% and an initial attendance of 50% at the OHS, we must invite 206 workers per group (103 bricklayers, 103 supervisors).

We do not expect major differences between the various offices of a single coordinating OHS. No restrictions or matching criteria were imposed.

### Measurements

Baseline data are obtained from the results of the job-specific WHS, CAU, the additional questionnaires to be filled in by the worker and OP and the official registration system for the OP. Two months after baseline, a follow-up measurement takes place; three months after baseline, a second follow-up measurement takes place. At all measurements occupation and any possible worker compensations such as early retirement or partial disability are queried.

Follow-up data are obtained from questionnaires to be filled in by the worker and the OP and the official registration system used by the OP to administer the planned and executed activities.

#### Primary outcome

The primary outcome variable (measured at the two-month and three-month follow-ups) compares the number of workers who have undertaken one or more preventive actions advised by their OP after attending their WHS relative to the total number of workers who attended their WHS. The outcome measure 'preventive action' is dichotomised into 'did undertake preventive action' for the workers who did make use of one or more adequate preventive actions and 'did not undertake preventive action' if no preventive action was undertaken.

Preventive actions are classified by the OP into the following domains: i) more detailed examination of the complaint or risk factor, for example, a visit to the OP, general practitioner or specialist, or a workplace visit); ii) individual preventive actions aimed at reducing risk factors or increasing work capacity (for example, visiting a physical therapist, participating in a lifestyle program) and iii) preventive actions taken at the technical or organisational level (for example workplace adjustments or training and education).

#### Secondary outcomes

Secondary outcome parameters are i) the proportion of workers with suggested preventive actions of the OP in their worker reportage (number of workers with one or more preventive actions in their report relative to the number of workers that attended their WHS); ii) the number and type of preventive actions (job-specific or general health) advised by the OP; iii) the number and type of preventive actions undertaken (job-specific or general health) by the worker and iv) work ability. Furthermore, the direct costs per worker for the funder of the WHS will be determined.

#### Process evaluation

In addition to the effect evaluation, the process of the intervention will be evaluated. The following components of the process will be evaluated: context, reach, dose delivered, dose received, fidelity and satisfaction [17].

Contextual aspects such as the political and economic environment may influence intervention implementation. Contextual factors will be monitored during data collection by questioning OPs and workers on economic recession, organisational or financial changes (for example, at the level of the OHS) and factors related to circumstances concerning the individual worker such as changes at the level of their employer.

Reach will be expressed as the attendance rate of the workers at their OHS. Attendance will be defined as the number of workers who attended their WHS relative to the number of workers who were invited by the OHS at baseline.

The dose delivered is a function of efforts of the OPs and other occupational health professional. Dose delivered will be defined as the number of preventive actions or follow-up arrangements provided (registered) to the worker by the OP at two or three months relative to the number of workers with an officially registered or noted follow-up appointment in the worker's report at baseline.

The dose received is the extent of engagement of the workers with the intervention. Dose received will be defined as the number of workers who undertake a prevention action at two or three months after their WHS relative to the number of workers who were advised to take a prevention action by the OP at baseline. In addition, we will assess the number of workers who intended to take a prevention action after their WHS at baseline relative to the number of workers who were advised to take a prevention action by the OP.

Fidelity is the extent to which an intervention is delivered as planned. We will assess the integrity and quality of the job-specific WHS by assessing protocol adherence. We developed performance indicators and will score these as 'sufficient' or 'insufficient' based on the workers' reportage and questionnaires at the two-month and three-month follow-ups. A total score will be calculated. Fidelity will be expressed as the frequency of total scores per group and per OP. Furthermore, we will ask the workers to what extent the WHS provided them more knowledge on their own health status and work ability.

The satisfaction of the workers with both CAU and the intervention will be evaluated in three ways. All workers will be asked to rate on a scale from 1 to 10 (not satisfied to very satisfied): 1) the WHS as a whole (at baseline); 2) the advised preventive actions (at baseline) and 3) the effect of the preventive actions on their health status and work ability (at the two-month and three-month follow-ups).

### Statistical analysis

The baseline data and the primary and secondary parameters will be presented using descriptive statistics. The following characteristics at baseline will be compared between the intervention and control groups: occupation, work experience in years in construction and in the current occupation, work hours per week and any possible worker compensations such as early retirement or partial disability. Furthermore, these characteristics will be compared overall and by study condition in those lost to follow-up and those retained. Moreover, the age and occupation of the study population at baseline and target population (all invited by the OHS) will be compared. Differences in continuous outcome measures will be tested with an independent samples T-test, differences in proportions will be tested with a Chi-Square test.

The effectiveness of the job-specific WHS on the primary and secondary outcome measures will be analysed on the individual level. First, data for the whole group will be analysed irrespective of the occupation of the worker. Second, subgroup analyses will be performed among the bricklayers and supervisors. For all analyses, a significance level of 0.05 will be applied. Workers with the same OP tend to be more homogeneous than workers with different OPs. Therefore, a two-level logistic regression model with workers at level 1 nested within OPs at level 2 will be used to analyse the binary data. The SPPS Advanced Statistics 19 software will be used to perform the logistic multilevel analysis by using a Generalised Linear Mixed Model (GLMM).

## Discussion

The results of this study will increase insight into the effectiveness of a job-specific WHS for construction workers in terms of the preventive actions undertaken by workers. The content of the job-specific WHS is based on evidence and is tailored to the specific demands of the occupation. By comparing the job-specific WHS to the current health surveillance program for construction workers, it will be determined whether a job-specific approach results in more preventive actions undertaken by both the OP and the worker. The evaluation process allows us to identify any key components of the job-specific WHS that are effective and under what conditions they are effective.

Although gains in health and work functioning are the ultimate goal of WHS, the health effects of participation in WHS are difficult to study because of several underlying reasons. For example, the health effects could occur years from now, they are likely to be small for several monitored health and they occur too infrequently to entail statistical changes with the small sample sizes used in this study. Therefore, we have chosen the behaviour of both the worker and the OP during and after the WHS as the outcome. The behaviour of both are a prerequisite for a successful surveillance program, because such programs can only show a preventive effect when action is taken.

### Strengths

Trials that are designed to evaluate a complex intervention often fail to provide useful information. An ineffective intervention might be the result of inadequate application or inappropriate context, and the results of an effective intervention might be difficult to generalise [18]. Evaluating a complex intervention (as in the present study) is therefore a considerable challenge. We believe we have undertaken several steps that are necessary in designing a useful evaluation trial. First, we have put a considerable amount of effort into developing a job-specific WHS for two construction occupations based on scientific evidence [3]. In addition to a systematic literature review, we conducted epidemiological research and a pilot trial. The pilot trial allowed us to explore expert opinions, to discover opportunities and barriers in implementing the job-specific WHS and to optimise its content.

Second, we have considered context as an important underlying factor in the success of an intervention. We acknowledge the unique occupational healthcare organisation for construction workers in the Netherlands. Therefore, we aimed to provide a clear view of the context of the Dutch situation and thereby to allow readers to determine the relevance of the present study to their situation.

Third, by choosing appropriate outcome measures, we stayed very close to the desired behavioural effects of the job-specific WHS to provide meaningful results. Furthermore, a thorough evaluation process allows a causal pathway of how and where the intervention failed to be identified.

### Limitations

The nonrandomised design of this study might be regarded as a limitation. We aim to contributing to evidence-based occupational health care, and in this context a randomised controlled trial is considered the gold standard to provide evidence for the effectiveness of an intervention. We considered randomisation at both the level of the individual worker and at the level of the OP, but we came across the practical limitations of a randomised trial. We felt that a nonrandomised design fit the best. In this way, we interfered as little as possible in the occupational health care routine for construction workers, which allowed us to evaluate the job-specific WHS in everyday practice.

We will perform subgroup analyses on the occupations, but we acknowledge the fact that our study cannot provide evidence of small differences. As we are dependent on the number of bricklayers and supervisors who are eligible for invitation by their OHS, the only possible solution would be to have more OHSs participating in the present study. In addition to the excessive extra costs, this solution would result in relatively few extra workers per OHS, as we already selected the largest offices of the OHS. Another obstacle is the economic recession, which results in a high percentage of dismissed workers in the construction industry.

The results of the present study will be available in 2013.

## List of abbreviations

CAU: Care As Usual; OHS: Occupational Health Service; OP: Occupational Physician; WHS: Workers' Health Surveillance.

## Competing interests

The authors declare that they have no competing interests.

## Authors' contributions

JB is responsible for data collection and drafted the manuscript. All authors conceived and designed the study, read and corrected draft versions of the manuscript and approved the final manuscript. HM, JS and MFD obtained funding for this study. JS and MFD were the co-principal investigators.

## Pre-publication history

The pre-publication history for this paper can be accessed here:

http://www.biomedcentral.com/1471-2458/11/743/prepub

## Supplementary Material

Additional file 1 **Development and content of the job-specific WHS**. Development and content of the job-specific workers' health surveillance for bricklayers and construction supervisors. This file describes the development and content of the job-specific workers' health surveillance for bricklayers and construction supervisors. Click here for file
