# Supplementary material for: A trial of a job-specific workers' health surveillance program for construction workers: study protocol
Source: BMC Public Health. 2011 Sep 29;11:743. doi: 10.1186/1471-2458-11-743 (PMC3192691; doi:10.1186/1471-2458-11-743)
Supplement: Additional file 1 — Development and content of the job-specific WHS. Development and content of the job-specific workers' health surveillance for bricklayers and construction supervisors. This file describes the development and content of the job-specific workers' health surveillance for bricklayers and construction supervisors. [file 1471-2458-11-743-S1.PDF]

Development and content of the job-specific workers' health surveillance for bricklayers and construction supervisors.

Additional file 1 by the manuscript 'A trial of a job-specific workers' health surveillance program for construction workers: study protocol'

Julitta S. Boschman[1], Henk F. van der Molen[1,2], Cor van Duivenbooden[2], Judith K. Sluiter[1] and Monique H.W. Frings-Dresen[1]

[1]Academic Medical Center, University of Amsterdam, Department: Coronel Institute of Occupational Health, Amsterdam, the Netherlands.
[2]Arbouw, Dutch Health & Safety Institute in the Construction Industry, Harderwijk, The Netherlands.

Content

Development of the job-specific WHS 3
Content of the job-specific WHS 3
Questionnaire 4
Biometry 8
Physical performance tests 8
Protocol to support the occupational physician 12
Pilot study 14

Development of the job-specific WHS

The Dutch Guideline for Workers' Health Surveillance for the Occupational Physician [1] was followed in the process of developing the job-specific workers' health surveillance (WHS) program. The content of the job-specific WHS was determined in a step-by-step procedure partially based on Bos et al. [15]. First, we conducted a systematic review of the occupational demands and health effects on bricklayers and supervisors [3]. For bricklayers, evidence was found for physical demands and for risk of low back pain and arms and leg complaints; for construction supervisors, psychosocial demands were identified. Both occupations are at increased risk of lung cancer and injury.

Second, we validated and supplemented the results from the systematic literature review by using information from expert documents and data from a questionnaire survey [unpublished data]. In this way, we gathered any additional or missing information on the demands and health effects of both occupations.

Third, we identified specific occupational job demands and matching job requirements. Fourth, proper measurement instruments were selected that sufficiently measure the job requirement and meet the criteria for screening [1].

Content of the job-specific WHS

In the job-specific WHS the following domains are represented: musculoskeletal system, safety (vision, perception of sound, psychological vigilance and working at heights), hazardous substances (skin, lungs), health in relation to work (cardiometabolic health) and work ability. To detect signals of work-related health problems, reduced work capacity or reduced work functioning, the worker fills in a questionnaire, then biometry measurements are carried out by a physician's assistant, and subsequently, physical performance tests are carried out under the guidance of an ergonomist. Thereafter, the OP discusses the results of

the job-specific WHS with the worker and suggests desirable preventive actions in a 20-minute consultation.

Questionnaire

The following screening instruments are included in the questionnaire:

Musculoskeletal system: The worker is asked “Did you have in the past six months regular complaints of joints, tendons or muscles?” (yes/no). If so, the worker is asked in which body region (neck, upper back, shoulder, elbow, forearm, wrist/hand, lower back, hip, knee, lower leg, ankle/foot), whether there is a restriction in doing his job (yes/no) and whether the complaint(s) are a consequence of the work (yes/no). Next, the bricklayers are asked about tasks relevant to their occupation “Do you currently have difficulties with… prolonged standing / working above shoulder height / prolonged working with a bent or twisted back / lifting and/or carrying blocks / picking up and laying bricks / working with a trowel / repetitive hand-arm movements / climbing stairs / climbing ladders or scaffolds / working at heights / maintaining balance under all circumstances” (yes/no). Construction supervisors are asked “Do you have problems with… climbing stairs / climbing ladders or scaffolds / working at heights / maintaining balance under all circumstances / walking across the construction site” (yes/no). A positive answer regarding regular complaints of the musculoskeletal system or difficulties with occupations-specific task performance are considered a signal.

Hand-arm vibration syndrome (HAVS): The worker is asked about symptoms of HAVS [19]: “When it’s cold, do you have attacks of paleness in at least one finger tip?” (yes/no) and “Do you have a numb or tingling feeling in one or more fingers?” (yes/no). A positive answer to one or both questions is considered a signal.

Hearing, sight: The worker is asked “Do you currently have difficulties with… sight (even when wearing glassed or lenses) / reading (even when wearing glassed or lenses) / sight when working in twilight / hearing your colleagues at the construction site / hearing

signals at the construction site"(yes/no). Furthermore, the worker is asked about symptoms: "Are you bothered by… weary or irritated eyes / hearing noises (peeping, rustling or ringing), not heard by others? (yes/no). A positive answer to one of the questions is considered a signal.

Safety: To screen for potential risks to the worker or others, the worker is asked: "Do you have sleeping problems?" (yes/no); "Are you awfully tired in recent weeks?"(yes/no); "Do you use medication or substances that have an effect on your work?" (yes/no). A positive answer to one or more of the questions is considered a signal.

Mental health: Several instruments are used to screen for mental health problems. The Need for recovery after work scale (Cronbach's alpha: 0.78) is used to measure work-related fatigue, which is assumed to be a link between repeated adverse work demands and the development of work-related stress reactions, (psychological) overload and, eventually, health problems [22,23]. Examples of the items on the Need for recovery after work scale are "At the end of a workday I'm exhausted" and "At the end of a workday I am really feeling worn-out". The items are answered with 'yes' or 'no'. More than six out of the eleven items indicating work-related fatigue is considered a signal.

Distress is measured with the Distress screener (Cronbach's alpha: 0.83), which is a valid tool for the early identification of distress in workers [24]. The questions are "During the past week, did you suffer from worry?'', "During the past week, did you suffer from listlessness?" and "During the past week, did you feel tense?" The items are answered with 'no' (0 points), 'sometimes' (1 point) or 'regularly or often' (2 points). The optimal cut-off-point to discriminate distressed workers was set at 4 points, for which the sensitivity is 0.80 and specificity 0.73.

Depression and anxiety are detected with the Dutch version of the Brief Symptom Inventory-depression (BSI-dep) (Cronbach's alpha 0.87) and BSI-anxiety scale (BSI-ang) (Cronbach's alpha 0.87). The BSI has been found to be a reliable and valid screening instrument [25,26]. The BSI-dep consists of 6 characterisations of depressed mood, for each of which the worker has to indicate how it applied to his/her situation during the last week

(including today) on a five-point Likert scale (0 = absolutely not; 4 = very much).The cut-off point for the scale score was set at ≥ 0.42. The BSI-ang also consists of six items that are answered and scored in the same way as the BSI-dep.

The Dutch version of the Impact of Event Scale is used to detect workers with a Post-Traumatic Stress Disorder (PTSD) [27]. A cut-off value of 26 on the Impact of Events Scale is used to distinguish the workers with a possible PTSD. The scale contains items such as “Any reminders brought back feelings about it”. The items are scored on a four-point Likert scale: never (0 points), rarely (1 point), sometimes (2 points), frequently (5 points). Exposure to a work-related, traumatic event was investigated by asking: “During the last year, have you witnessed or experienced a (near) accident at the construction site?” (yes/no).

Alcohol consumption is measured with the first three questions of the Alcohol Use Disorder Test Consumption (AUDIT-C) [28]. The derived AUDIT-C score is calculated from responses to the following three questions for all types of alcohol combined: (1) overall frequency of drinking, (2) usual quantity of drinks consumed, and (3) frequency of drinking ≥6 drinks at the same occasion. The derived AUDIT-C performs well in screening for alcohol use disorders and risky drinking [28]. Risky drinking is defined as exceeding the cut-off value of 5 points or exceeding recommended daily drinking limits once a month or more often.

For construction supervisors, the questionnaire is supplemented with an adapted version of the Utrechtse Burnout Schaal (UBOS), the Dutch translation of the Maslach Burnout Inventory [29]. The Dutch translation of the MBI consists of three scales: emotional exhaustion (8 items), depersonalisation (5 items) and personal accomplishments (7 items) (Cronbach’s alpha 0.89, 0.67 and 0.75, respectively). The scoring ranges from 0 “never” to 6 “every day”. High levels of emotional exhaustion and depersonalisation are characteristic of burnout and are used as screening instrument. The emotional exhaustion and depersonalisation scales are able to discriminate between burned out and nonburned out workers. The cut-off points are set at 21 points (emotional exhaustion) and 10 points (depersonalisation). We replaced generic terms such as ‘persons’ or ‘other people’ with

‘personnel’, a relevant, job-specific operationalisation of the persons a construction supervisor is involved with.

Skin: Bricklayers are screened with a questionnaire on occupational hand dermatitis that is appropriate for use as a screening instrument due to it’s high sensitivity (100%) [30]. Furthermore, for the bricklayers the OP carries out a dermatological examination of the skin of the fingers, hands and arms to assess symptoms of hand dermatitis. Both bricklayers and construction supervisors are questioned about other skin problems indicative of skin cancer: “Do you have on your skin… spots that change colour, shape or thickness / itchy or bleeding spots / birthmarks, old scars or ulcers that are healing with difficulty?” (yes/no).

Respiratory system: The worker is asked “In the past six months, did you have any breathing or respiratory complaints?” (yes/no). A positive answer is considered a signal and factor to be addressed by the OP, but in conjunction with the results of spirometrical assessments of symptoms of Chronic Obstructive Pulmonary Disease or asthma.

Risk factors cardiometabolic diseases: In order to detect an increased risk of cardiovascular disease (CVD), type 2 diabetes and chronic kidney disease a risk score based on age, body mass index, waist circumference, smoking behaviour and family history of diabetes and CVD is calculated [32]. Furthermore, the worker is asked about having a previous history of cardiovascular disease, having type 2 diabetes and symptoms of CVD (shortness of breath, pain or oppressed feeling in the chest or cardiac region). Furthermore, levels of blood glucose and cholesterol are measured in order to compile a cardiovascular risk profile [33].

Work ability: The first questions of the Work Ability Index (WAI) are used to measure current work ability, work ability in relation to the physical and mental work demands, current chronic disorders and limitations due to these disorders [34-36]. The instrument is used to provide the OP with relevant and useful information about the work ability of the worker.

Biometry

A physician's assistant assesses the worker's eyesight at 5 m [21] hearing by means of an audiogram [20], respiratory function by means of standard spirometry [31], weight and height to calculate body mass index, waist circumference, blood pressure, blood glucose and cholesterol [33].

Physical performance tests

Physical capacity is measured during physical performance tests resembling physically demanding working situations. The physical tests were designed in such a way that body parts are tested in both an isolated manner and in more complex movements with several types of load combined. The intensity, frequency and duration of the functional tests were based on information from the literature, expert knowledge and the Dutch Occupational Health and Safety Act. For bricklayers, the measured, estimated or maximally allowed load for a full work day is converted to a 90-minute test protocol. Both bricklayers and supervisors are observed while climbing a 7 m ladder once and walking one flight of stairs up and down six times. Supervisors then walk for 5 min in a pace of approximately 5 km/h and repeat the climbing of stairs en walking once, so that they climb a total of 12 stairs en walk for 10 minutes in total.

The bricklaying test for bricklayers consists of three parts that are repeated twice: 1) isolated repetitive hand-arm movements; 2) picking up and laying of bricks and blocks and 3) lifting and carrying 25 kg bags. The total duration of the bricklaying test protocol is 90 minutes, including instructions and clearing of the test setting. During the first part the bricklayer performs in five minutes: a) one minute of sand mixing (Figure 1); b) a total of three minutes scooping sand from one bucket to another for one minute at each of three levels (from ground level to ground level, from ground level to 50 cm and from ground level to 100 cm). During each minute, approximately 25 kg of sand is transferred. The last

component is c) repetitively (50 times a minute) rotating the wrist from maximal supination to maximal pronation with an 0.9 kg trowel for one minute.

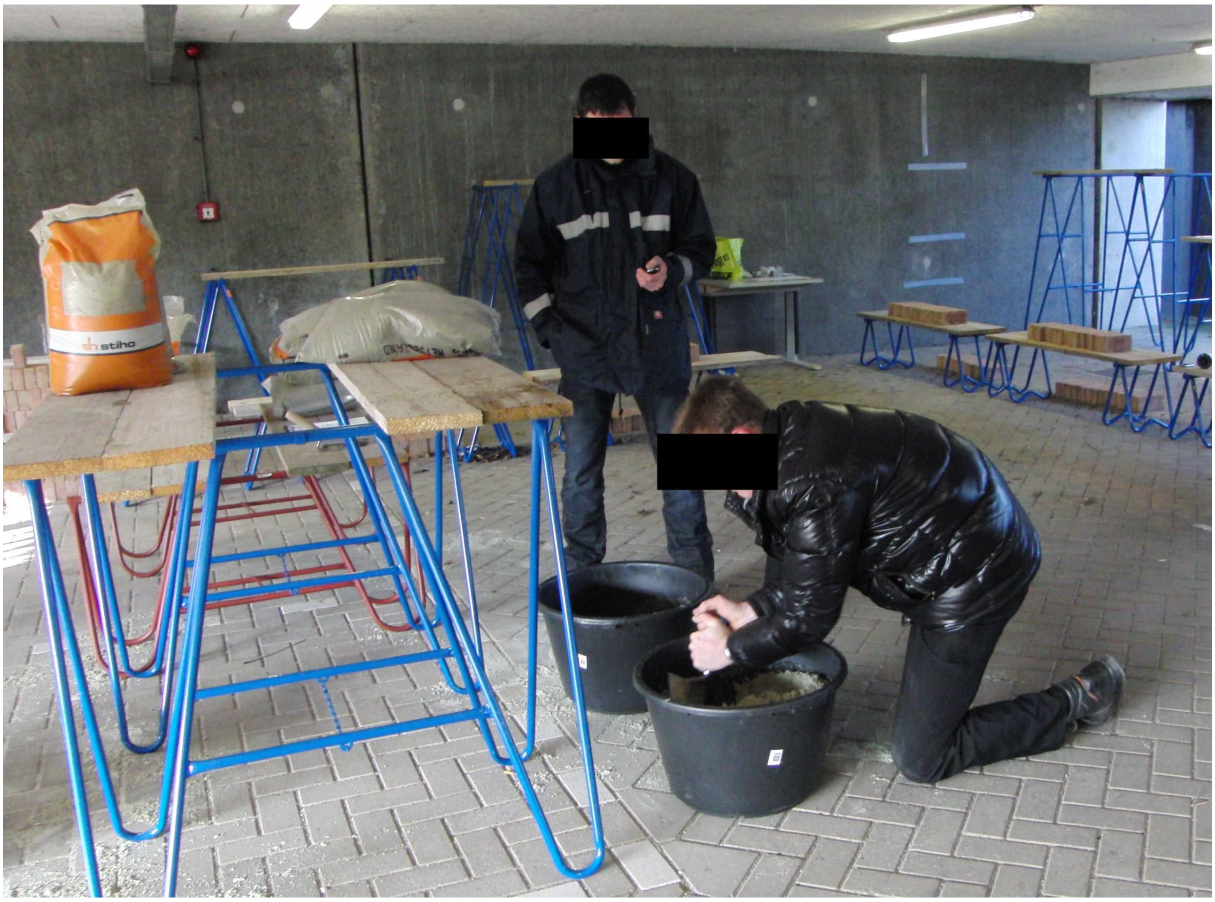

Figure 1. Mixing of mortar-like sand.

During the second part, the bricklayer has to pick up bricks and build a wall for 16 minutes in a self-chosen work pace. For eight minutes the bricklayer has to work from ground level to ground level (Figure 2). Next, the bricklayer works another eight minutes by picking up the bricks from 45 cm and placing them at ground level (Figure 3). Picking up of mortar is simulated by using an 0.9 kg trowel, and the bricklayer is instructed to make a wall approximately 1.5-2 m wide and not higher than five bricks. During the first four minutes the bricklayer works in a self-chosen position. Thereafter, he is asked to work for two minutes in a kneeling position, two minutes in a crouching position and two minutes in a bent position.

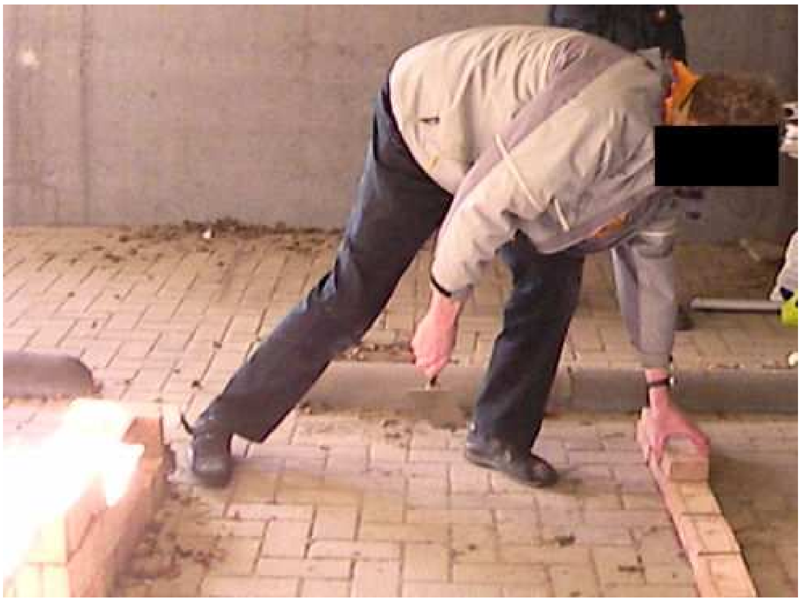

Figure 2. Laying bricks from ground to ground level.

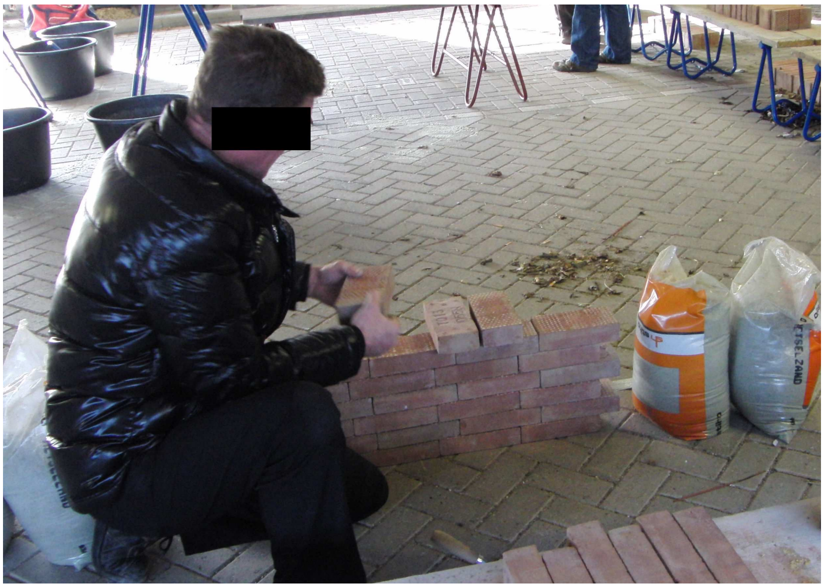

Figure 3. Laying bricks from a 45 cm support to ground level

The third part consists of lifting and carrying eight 25 kg bags. The bags are picked up from the ground and carried for ~3-3.5 m. Four bags are then placed at 50 cm and four bags are placed at 100 cm.

After the third part, the first part is repeated in the same way. Thereafter, the second part is repeated at different heights. The bricklayer then has to work for 24 minutes according to the following scheme at self-chosen pace and a self-chosen posture:

four minutes: picking up from the ground, laying at 50 cm;

four minutes: picking up from a 45 cm support, laying at 50 cm;

four minutes: picking up blocks from the ground (two-handed), laying at 100 cm;

four minutes: picking up blocks from a 45 cm support (two-handed), laying at 100 cm;

four minutes: picking up from the ground, laying at 150 cm;

four minutes: picking up from a 45 cm support, laying at 150 cm (Figure 4).

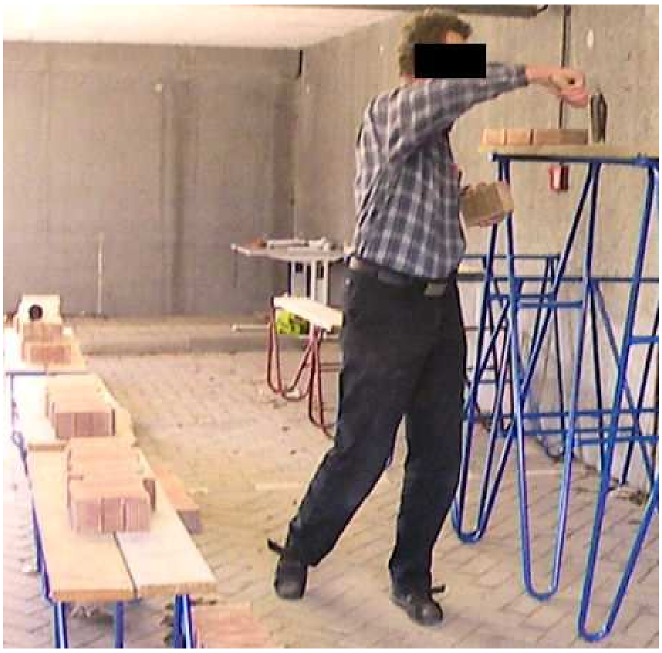

Figure 4. Laying bricks from a 45 cm support to 150 cm.

Next, the third part is repeated, but now, the bags are transferred in the opposite direction. The bags are picked up from 50 cm and 100 cm, carried for 3-3.5 m and placed at ground level.

During the physical performance tests, the ergonomist observes the worker and notes possible limitations and/or compensations in the execution of the task. The ergonomists asks the worker after every part of the test whether they have experienced any discomfort, pain or restrictions. The ergonomist asks the bricklayer about the following risk factors and their occurrence during a regular work day: lifting (asymmetrical, heavy), deep bending, bending and twisting, kneeling, working with raised/tensed shoulders, working and lifting above shoulder height, little variation in working posture, unilateral physical stress. On the basis of the observations and findings during the tests, the ergonomist makes, if necessary, a recommendation to the OP concerning possible preventive actions such as a workplace visit, work place adjustment, another working method or (temporarily) adapted working and resting hours.

Protocol to support the occupational physician

For the OP, a structured protocol for providing advice and counselling on job-specific intervention measures was developed. The aim of the protocol is to facilitate the preventive action recommendations by the OP. The OP composes a report for their own use and that of the worker. Based on the results and their priority the OP determines desirable preventive actions to be implemented first. Preventive actions for both occupations are categorised into the following categories: i) a more detailed examination of the complaint or risk factor (for example a visit to the OP, general practitioner or specialist, or a workplace visit); ii) advising and counselling on individual preventive actions aimed at reducing risk factors or increasing work capacity (for example, visiting a physical therapist, participating in a lifestyle program) and iii) preventive actions taken at the technical or organisational level (for example, workplace adjustments or training and education).

The OP discusses the results of the job-specific WHS and suggests desirable preventive actions to the worker in a 20-minute consultation. Together with the worker, a

course of action is drawn up and a follow-up appointment is arranged within eight weeks after the WHS, if necessary.

Pilot study

Finally, we conducted a pilot study in which we determined the feasibility of implementing the job-specific WHS in terms of acceptability and practicability. The pilot study was conducted at one office of an OHS. Ten bricklayers, 16 supervisors, two OPs, three physician's assistants and two ergonomists participated in the study. The main purpose of the pilot study was to verify the practicability and suitability of the functional tests and the questionnaire. All participants filled in a questionnaire with several topics related to the feasibility of and their satisfaction with the job-specific WHS. Furthermore, all participants were interviewed in a semi-structured way.

From the experiences of the participating workers and occupational health professionals, we made minor adjustments to the physical performance tests in order to have them resemble working situations more closely. Furthermore, the OPs indicated they would need training to carry out the job-specific WHS in accordance with the protocol. Because of uncertainties regarding the safety of the participants when climbing a scaffold, expressed by both the participants and the ergonomists, we replaced the climbing of a scaffold with climbing a ladder.
